# Investigating genetic profiles of cases of *Schistosoma* spp. imported into Europe: a cohort from the European Society of Clinical Microbiology and Infectious Diseases Study Group for Clinical Parasitology

**DOI:** 10.1186/s13071-025-07164-5

**Published:** 2025-12-15

**Authors:** Elena Pomari, Bonnie L. Webster, Elena Locatelli, Miriam J. Álvarez-Martínez, Marta Arsuaga, Emmanuel Bottieau, Olivier Bouchaud, Daniel Camprubi-Ferrer, Maura Concu, Rosa de Miguel Buckley, Rob Koelewijn, Davide Marangoni, Anthony Marteau, Beatrice Nickel, Camilla Rothe, Fernando Salvador, Mirjam Schunk, Lidia Goterris, Marjan Van Esbroeck, Jaap J. van Hellemond, Linda J. Wammes, Lorenzo Zammarchi, Sofia Pettene, Eleonora Rizzi, Salvatore Scarso, Federico G. Gobbi, Francesca Tamarozzi

**Affiliations:** 1 Department of Infectious-Tropical Diseases and Microbiology, IRCCS Sacro Cuore Don Calabria Hospital, 37024 Negrar Di Valpolicella, VR Italy; 2 Department of Science, The Natural History Museum, London, SW75BD UK; 3 Microbiology Department, Hospital Clínic, University of Barcelona, 08036 Barcelona, Spain; 4 Barcelona Institute for Global Health (ISGlobal), 08036 Barcelona, Spain; 5 National Referral Unit for Imported Tropical Diseases and Health Travel, Hospital La Paz-Carlos III, 28034 Madrid, Spain; 6 Centro de Investigación Biomédica en Red de Enfermedades Infecciosas (CIBERINFEC), Instituto de Salud Carlos III, 28029 Madrid, Spain; 7 Department of Clinical Sciences, Institute of Tropical Medicine, 2000 Antwerp, Belgium; 8 Department of Infectious and Tropical Diseases, Avicenne Hospital, AP-HP, 93000 Bobigny, France; 9 CEPED-Sorbonne Paris Nord University, Bobigny, France; 10 International Health Department, Hospital Clínic, 08036 Barcelona, Spain; 11 Swiss Tropical and Public Health Institute, 4123 Allschwil, Switzerland; 12 University of Basel, 4001 Basel, Switzerland; 13 Department of Medical Microbiology and Infectious Diseases, Erasmus MC University Medical Center, 3015 Rotterdam, The Netherlands; 14 Dipartimento di Medicina Sperimentale e Clinica, Università degli Studi di Firenze, 50134 Florence, Italy; 15 Parasitology-Mycology Department, Sorbonne Paris Nord University, AP-HP, Avicenne Hospital, 93000 Bobigny, France; 16 Institute of Infectious Diseases and Tropical Medicine, Ludwig-Maximilians-Universität (LMU) University Hospital, 80539 Munich, Germany; 17 International Health Unit Vall d’Hebron-Drassanes, Infectious Diseases Department, Vall d’Hebron University Hospital, PROSICS Barcelona, 08035 Barcelona, Spain; 18 Microbiology Department, Vall d’Hebron University Hospital, PROSICS Barcelona, 08035 Barcelona, Spain; 19 Center for Infectious Diseases (LUCID), Leiden University Medical Center, 2333 ZA Leiden, Netherlands; 20 Struttura Organizzativa Dipartimentale Malattie Infettive e Tropicali, Azienda Ospedaliero-Universitaria Careggi, 50134 Florence, Italy; 21 Department of Clinical and Experimental Sciences, University of Brescia, 25121 Brescia, Italy

**Keywords:** *Schistosoma* species, Schistosomiasis, Genetic profiles, Imported infections, Travellers, Migrants, Europe

## Abstract

**Background:**

The potential of schistosomiasis to spread across borders, coupled with the considerable delay by which infected travellers and migrants are diagnosed in Europe, calls for better surveillance of the distribution of this disease. This study explored the geographical origin and genetic profiles of *Schistosoma* infections imported into Europe and diagnosed in a network of 11 European centres specialized in traveller and migrant health.

**Methods:**

Genetic profiles were obtained from DNA extracted from concentrated *Schistosoma* eggs or *Schistosoma*-positive samples (faeces, urine, biopsy) collected during routine diagnostic procedures. The species-specific cytochrome oxidase sub-unit 1 (*cox1*) diagnostic region and the standard complete internal transcribed spacer (ITS) 1 + ITS2 (ITS1 + 2) ribosomal DNA region were amplified and sequenced, together with a partial region of 18S ribosomal DNA in selected cases. Prevalences of the different genetic profiles within the whole patient cohort and by country/geographical area of possible infection were analysed. A phylogenetic analysis was performed using the larger *cox1* (~ 956 base pairs) sequences dataset.

**Results:**

A total of 94 samples were available for analysis, 36 from patients with a diagnosis of intestinal schistosomiasis and 58 with urinary schistosomiasis, all acquired in a sub-Saharan African country. Mitochondrial (mt) *cox1*, nuclear ITS1 + 2 and/or 18S (mt/nuclear) genotypes were successfully obtained from 51/94 (54%) samples; while for 43/94 (46%) samples, only a partial mt genotype was obtained. Infections with *Schistosoma haematobium* and *Schistosoma mansoni* were identified in the majority of cases (66/94; 70%), while mixed *Schistosoma* spp. genetic profiles, which were identified in 30% (28/94) of the samples, were almost exclusively (27/28; 96%) associated with cases of urinary schistosomiasis. Among the urinary infections, almost half (27/58; 47%) could be identified as having a mixed genetic profile. These mostly (26/28; 93%) included genetic traits of *S. haematobium* and *Schistosoma bovis*, and all were from patients probably infected in West Africa.

**Conclusions:**

Infections with *S. haematobium* and *S. mansoni* represent the majority of cases of schistosomiasis currently being diagnosed in Europe; however, mixed *Schistosoma* genetic profiles (mostly *S. haematobium/S. bovis*) were identified in at least 30% of samples. Our results call for a coordinated effort encompassing prompt diagnosis and treatment of *Schistosoma* infections, together with monitoring of the possible introduction of species of *Schistosoma* and establishment of their autochthonous transmission under suitable conditions in Europe.

**Supplementary Information:**

The online version contains supplementary material available at 10.1186/s13071-025-07164-5.

## Background

Schistosomiasis, which is caused by infection with dioecious trematodes of the genus *Schistosoma*, affects ~ 250 million people in countries endemic for this disease, of whom over 85% reside in sub-Saharan Africa [1]. Chronic schistosomiasis, caused by the inflammation elicited around parasite eggs entrapped in organs and tissues, encompasses urogenital and intestinal-hepatosplenic diseases, and is responsible for an estimated 2.5 million disability-adjusted life years and considerable mortality [2, 3]. The clinical manifestations of urogenital schistosomiasis range from haematuria, urinary symptoms and pelvic pain, to obstructive uropathy, bladder cancer, genital complaints and reproductive issues, while those of intestinal-hepatosplenic schistosomiasis include abdominal pain, hepatosplenomegaly, portal hypertension, and pulmonary hypertension [3, 4].

*Schistosoma* trematodes have a life cycle dependent on a freshwater environment, where intermediate snail hosts are infected by miracidia hatched from eggs released with urine or faeces of the infected definitive vertebrate host. In the snail, larvae multiply and develop until cercariae are released into the water, which can infect definitive hosts through percutaneous penetration. Eventually, adult male and female worms develop, pair and produce eggs, in venules in various parts of the body. The genus *Schistosoma* comprises 23 species, grouped into six clades of sister species, each with a more or less broad range of intermediate and definitive hosts (reviewed in [5]). In humans, the classic cause of urogenital schistosomiasis is *Schistosoma haematobium*, which is endemic in Africa (with cases also reported in the Arabian Peninsula), while intestinal and hepatosplenic disease is caused by five other species: *Schistosoma mansoni* in Africa and South America (with endemicity also known in the Arabian Peninsula and the Caribbean)*, Schistosoma intercalatum* and *Schistosoma guineensis* in Africa, and *Schistosoma japonicum* and *Schistosoma mekongi* in Asia [3]. Schistosomiasis is the second most prevalent neglected tropical disease of migrants who travel to Europe [6]; however, epidemiological data such as those on infecting species and population genetics are scant and not routinely gathered in a diagnostic/clinical setting.

Co-endemicity of *Schistosoma* species can result in co-infections, enabling inter-species interactions. The dynamics and outcomes of these interactions depend on the endemic setting and the species involved [5]. Molecular tools used to genetically characterize African *Schistosoma* populations have revealed complex human infections, with inter-species interactions enabling inter-species genetic exchange via hybridization [7, 8]. The occurrence of hybridization between certain *Schistosoma* species has been suspected since the beginning of the past century, based on phenotypical observations [9]. With the introduction of basic molecular genotyping techniques in the 2000s, inter-species *Schistosoma* hybridization was identified in sympatric African foci between species that infect humans (e.g. *Schistosoma haematobium* × *Schistosoma intercalatum* and *Schistosoma haematobium* × *Schistosoma mansoni*) and species that infect animals (e.g. *Schistosoma bovis* × *Schistosoma curassoni*), and also between *Schistosoma* species that infect humans or animal hosts (e.g. *Schistosoma haematobium* × *Schistosoma bovis* or *Schistosoma haematobium* × *Schistosoma mattheei*) [9], raising questions regarding *Schistosoma* species host specificities in Africa and risk of zoonoses.

More recent in-depth genomic analyses tracked backe some hybridization events (namely between *S. haematobium* × *S. bovis*) that resulted in genomic introgression as far as 240 years ago (range 107–612 years ago), with conventional hybridization considered to be rare [10–13]. The relevance of *Schistosoma* co-infections and infection with hybrids/introgressed forms for clinical aspects of the disease in natural settings is still not clear, and thus far, no differences in treatment outcomes have been observed [14–16] and robust conclusions could not be drawn from pathological investigations [15, 16]. One aspect of inter-species *Schistosoma* hybridization/introgression that would give a transmission advantage is related to snail intermediate host compatibility. Experimental studies have clearly shown that hybrid progeny inherit snail compatibility traits from both their parental species [9, 17, 18]. Therefore, there is a high potential for geographical range expansion via increased snail host compatibility, coupled with the frequent movement of livestock and people who may carry the infections [9, 10, 19–22].

Recent reports of autochthonous transmission of urinary schistosomiasis in Spain and Corsica [11, 23–28] highlight the ability, when given the opportunity, of *Schistosoma* species to colonize new environments, even in countries with high standards of hygiene. Such scenarios are supported by the combination of endemicity of permissive *Bulinus* snails (namely *Bulinus truncatus*) in these areas [18], urinary egg excretion (which is not easy to control) and recreational contact with freshwater. The role of genetically diverse *Schistosoma* populations (i.e. hybrids and introgressed forms) that cause urinary schistosomiasis, although not the sole driver, may further support such adaptations.

The potential of schistosomiasis, in particular urinary schistosomiasis, to spread across borders, coupled with the considerable delay by which infected travellers and migrants are diagnosed in Europe—leading to health consequences and potential epidemiological risks [29, 30]—and the possible change in the distribution of snail hosts driven by climate change [31, 32], call for better surveillance of the introduction of schistosomiasis and its distribution. The results of this study add to those of previous ones [6, 15, 33] on cases of imported schistosomiasis which examined the infecting species, their genetic makeup and geographical origin. The results used in the present study were obtained by capitalizing on the work of a network of European centres that specializes in traveller and migrant health and uses a whole-sample analysis approach, providing data on a wide spectrum of imported cases across Europe.

## Methods

### Study design and objectives

A cross-sectional analysis was carried out of concentrated *Schistosoma* eggs or *Schistosoma*-positive samples collected during routine diagnostic procedures for schistosomiasis or that had been prospectively collected between April 2021 and March 2024 in the participating centres. The study objectives were to identify and genetically characterize the *Schistosoma* infections from migrant and traveller patients, and to map their distribution according to the geographical origin of the infection.

### Study population and data collection

Clinical samples included faeces (concentrated by sedimentation without formalin, or unprocessed), biopsies, concentrated eggs from urine (from sedimentation or filtration), or DNA extracted from these types of clinical samples. Samples were eligible for inclusion in the study if they had been stored frozen or preserved in ethanol (excluding urine) and the patient’s country of birth and/or most likely country/countries of infection were known from the patient’s medical records. Other data retrieved from the medical records, whenever available, were the patient’s sex and age, and *Schistosoma* species identification via microscopy and/or polymerase chain reaction (PCR).

### Molecular analyses

DNA from concentrated eggs from urine samples and from faecal samples (approximately 2 mg) was extracted using the DNeasy Blood & Tissue Kit (Qiagen, Hilden, Germany) and the QIAamp DNA Stool Mini Kit (Qiagen), respectively. Briefly, frozen samples were thawed, mixed with lysis buffer and proteinase K, with the addition of impurity removal buffer for faecal samples, and mechanically disrupted with ceramic bead-beating (MagNA Lyser; Roche, Basel, Switzerland). DNA was then purified from each sample using the standard methodology for the DNeasy Blood & Tissue Kit (Qiagen) and the QIAamp DNA Stool Mini Kit (Qiagen), with the DNA eluted in 50–100 µl Buffer AE. All of the DNA samples were stored at −20 °C for further molecular analysis.

Genetic profiles were obtained from each sample using a multi-locus [mitochondrial (mt) and nuclear DNA] approach, as described previously [34–37] (Supplementary Figure S1, Table S1). Firstly, attempts were made to characterize all of the samples by amplifying and sequencing the species-specific cytochrome oxidase sub-unit 1 (*cox1*) diagnostic region together with the standard complete internal transcribed spacer (ITS) 1 + ITS2 (ITS1 + 2) ribosomal DNA (rDNA) region. If the ITS1 + 2 rDNA region could not be amplified and/or genetic signatures of bovid *Schistosoma* species other than *S. bovis* were expected, a partial region of 18S rDNA was amplified and sequenced. Additionally, a generic *Schistosoma* PCR was included to generate a large *cox1* fragment in order to perform the phylogenetic analysis.

### Mitochondrial *cox1* genetic profiling

Samples were first analysed using a diagnostic multiplex PCR that produces amplicons of different size for the different species [*S. haematobium* ~ 543 base pairs (bp), *S. bovis* ~ 306 bp, *S. mansoni* ~ 375 bp] (Supplementary Table S1). PCRs were performed in 25-µl reactions containing 5 µl of each genomic DNA extract, HotStarTaq Master Mix (Qiagen) and 400 nM of each forward and reverse primer, and run with positive (*S. haematobium* and *S. mansoni* genomic DNAs) and negative (no template control) controls. The PCR cycle was 15-min denaturing at 95 °C: followed by 40 cycles of 30 s at 94 °C, 30 s at 58 °C, 1 min at 72 °C; followed by a final extension period of 10 min at 72 °C. PCR products (5 µl) were visualized on a 1.5% agarose gel and the amplicon sizes were determined by using a DNA ladder. All samples showing a clear specific single band were selected for further sequencing analysis, following purification.

A second *cox1* PCR was performed to amplify a larger fragment (~ 956 bp) to facilitate phylogenetic analyses (Supplementary Table S1). The PCR reaction mix was as described above with the following PCR cycling conditions: 15 min denaturing at 95 °C; 40 cycles of 30 s at 94 °C, 30 s at 58 °C, 1 min at 72 °C; followed by a final extension period of 10 min at 72 °C for the first PCR, and 15 min denaturing at 95 °C; 40 cycles of 30 s at 94 °C, 30 s at 40 °C, 1 min at 72 °C; followed by a final extension period of 10 min at 72 °C. PCR products (5 µl) were visualized on a 1.5% agarose gel and the amplicon sizes determined using a DNA ladder. All samples showing a clear specific single band were selected for further sequencing analysis, following purification.

### ITS1 + 2 rDNA and 18S genetic profiling

ITS1 + 2 PCRs (Supplementary Table S1) were performed using the same PCR reaction mix as described above with the following PCR cycling conditions: 15 min denaturing at 95 °C; 40 cycles of 30 s at 94 °C, 30 s at 45 °C, 1 min and 30 s at 72 °C; followed by a final extension period of 10 min at 72 °C. When no ITS1 + 2 results were detected or were inconclusive, a partial region of the 18S rDNA (~ 289 bp) [36] was analysed by using the same PCR reaction mix as described above and the following PCR conditions: 15 min denaturing at 95 °C; 40 cycles of 30 s at 94 °C, 30 s at 50 °C, 1 min at 72 °C; followed by a final extension period of 10 min at 72 °C. All PCRs were visualized on a 1.5% agarose gel. All samples showing a clear specific single band were selected for further sequencing analysis following purification.

### Sequencing and data analysis

All positive PCR reactions were purified using the ExoSAP-IT PCR Product Cleanup Reagent (Thermo Fisher Scientific, Waltham, MA) according to the manufacturer's protocol. Amplicons were Sanger sequenced in both the forward and reverse direction using a dilution of the original PCR primers. Sanger sequencing reactions were performed using BigDye version 3.1 Cycle Sequencing reagents (Thermo Fisher Scientific) and were run on an Applied Biosystems 3500 automated sequencer. For each amplicon, forward and reverse sequences were assembled and manually edited using BioEdit Sequence Alignment Editor (version 7.2.5). Sequences were trimmed to remove sequencing errors at the ends of the amplicons and all sequence ambiguities were checked by visualization of the sequence chromatograms. The quality of the sequences was also assessed by visualisation of the sequence chromatograms.

For the *cox1* data, a contig was formed from the forward and reverse sequences from which a consensus sequence was created. Identification of the *cox1* consensus sequences for each sample/amplicon was confirmed using the Basic Local Alignment Search Tool (BLAST 2.15.0 with megablast option).

The ITS1 + 2 forward and reverse sequences were aligned to create a contig. This was then aligned with the reference ITS1 + 2 for *S. haematobium* (MT580953.1), *S. bovis* (PP312935.1), *S. curassoni* (MT580947.1), *S. mattheei* (Z21718), and *S. guineensis* (Z21727) available from GenBank [35, 36]. The sequence chromatograms for each sample were analysed with a specific focus on the species-specific single nucleotide polymorphisms (SNP) for each *Schistosoma* species. SNP profiles were created for each sample to identify the ITS1 + 2 genotype. Occurrences of double chromatogram peaks at the species-specific SNP sites were also recorded to identify mixed ITS genotypes. Additional analysis of partial region of the 18S rDNA was performed for selected samples and the species-specific SNPs analysed after alignment with reference sequences AY157238.1 *S. bovis*, Z11976.1 *S. haematobium*, AY157236.1 *S. curassoni* and AY157235.1 *S. guineensis* (accessioned as *S. intercalatum*) available from GenBank, to enable sequence identity.

Mitochondrial (*cox1*) and nuclear (ITS1 + 2 and 18S) genetic profiles were compiled for each sample to provide an overall genetic profile for each sample. The data were further analysed with respect to prevalences of the different genetic profiles within the whole patient cohort and by country/geographical area of origin.

### Phylogenetic analysis

The phylogenetic analysis was performed using the larger *cox1* (~ 956 bp) sequences dataset available from 38/94 (41%)  of the study samples. The GenBank reference sequence of S. *haematobium* NC_008074.1 was included in the analyses and *S. curassoni* OX104147.1 was used as an outgroup. We also used sequences of *S. haematobium* and *S. bovis* to verify and confirm what was obtained from the analysis of the Sanger sequencing and *S. bovis* mitotype sequences of *S. haematobium* × *S. bovis* hybrids to support species identification. A quality control of the sequences was carried out, followed by multiple sequence alignment using Clustal Omega (version 1.2.4). We performed the trimming of sequence ends followed by trimAl (version 1.5.rev0), adopting a gap threshold of 50%, to provide uniform ends. Maximum likelihood was used as the statistical method. To assess the reliability of the nodes in the tree, a bootstrap analysis using 1000 replicates was undertaken. The phylogenetic tree was build using IQTREE program from command line (iqtree version 2.3.5) and iTOL (version 7.1).

## Results

### Demographic and routine parasitological data

**Fig. 1A, B:**
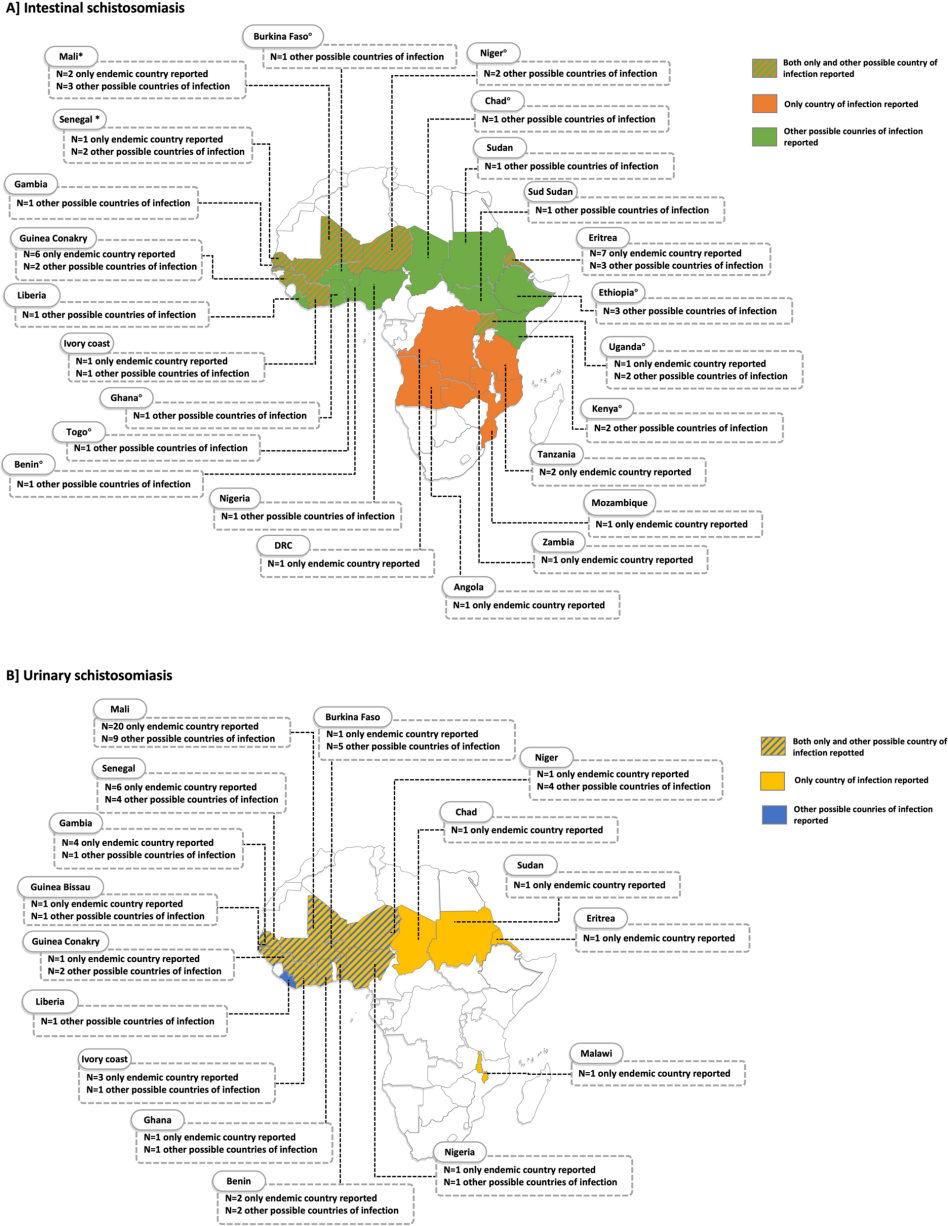
Reported possible country/countries of infection with *Schistosoma* spp. Numbers represent the number of patients reporting possible infection in the given country. **A** Patients clinically diagnosed with intestinal schistosomiasis. Areas indicated in orange show the countries of possible infection for patients reporting exposure in one country only. Areas indicated in green show the countries of possible infection for patients reporting possible infection in multiple countries. Areas indicated by green-orange stripes show the countries of possible infection for some patients reporting infection in that country only and for other patients having had possible exposure in multiple additional countries. All infections at diagnosis were identified as caused by *Schistosoma mansoni* from the analysis of stool (microscopy or polymerase chain reaction), with the exception of six patients for whom species identification was not conclusive. One of these patients (who visited countries indicated by a degree symbol), who was born in Australia and in whom *Schistosoma* spp. eggs were found in a biopsy of the appendix, reported having travelled in Benin, Burkina Faso, Chad, Ethiopia, Ghana, Kenya, Niger, Togo and Uganda. Additionally, in one patient from Mali and one patient from Senegal (who had visited countries indicated by an asterisk) eggs with a shape typical of those of *Schistosoma haematobium* were identified in the stool, and for one patient from Senegal, who had also travelled in Mali, eggs with a shape typical of those of *Schistosoma intercalatum* were identified in the stool. **B** Patients diagnosed clinically with urinary schistosomiasis. Areas indicated in yellow show the countries of possible infection for patients reporting exposure in one country only. Areas indicated in blue show the countries of possible infection for patients reporting possible infection in multiple countries. Areas indicated by blue-yellow stripes indicate the countries of possible infection for some patients reporting infection in that country only and other patients having had possible exposure in multiple other countries. All infections but one (which was not identified to the species level) were identified at diagnosis as caused by *S. haematobium.* The map was produced using www.yourfreetemplates.com. Individual patient’s data are available in Supplementary Table S2. *DRC* Democratic Republic of the Congo

A total of 94 eligible samples were available for analysis (Table 1; Supplementary Table S2). The majority (83/94; 88%) of the patients were males and the median age of the cohort was 21 years (range 8–48). The country of birth was available for 89 patients: 82/89 (92%) patients were born in a sub-Saharan African country, most commonly Mali (*n* = 22; 24%), followed by Senegal (*n* = 10; 11%); only seven patients (8%) were non-African travellers, and were from Belgium (*n* = 2), Australia, Germany, Japan, Poland and Spain (*n* = 1 each). In all cases, the origin/s of possible infection was a sub-Saharan African country (Fig. 1).

**Table 1:** Demographic data and initial diagnosis of schistosomiasis of the patients included in the study

	Total (*n* = 94)	Intestinal schistosomiasis (*n* = 36)	Urinary schistosomiasis (*n* = 58)
Sex [male (M), female (F)] (*n*]	M = 83, F = 11	M = 27, F = 9	M = 56, F = 2
Age [median (range)] (years)	21 (8–48)	26 (8–47)	19 (8–48)
*Schistosoma* species identified at diagnosis	*Schistosoma haematobium, n* = 59; *Schistosoma mansoni*,* n* = 28; *Schistosoma intercalatum*,* n* = 1; not identified,* n* = 6	*S. mansoni*,* n* = 28; *S. haematobium, n* = 2; *S. intercalatum*,* n* = 1; not identified,* n* = 5	*S. haematobium*, n = 57; not identified,* n* = 1

Thirty-six patients had a diagnosis of intestinal schistosomiasis, based on identification of *Schistosoma* eggs in faecal samples by microscopy and/or *Schistosoma* DNA detected by PCR (Supplementary Table S2). Eggs with a shape typical of those of *Schistosoma mansoni* were identified in 28/36 (78%), those typical of *S. haematobium/S. guineensis* (atypical for intestinal schistosomiasis) in two (5%) cases, and those typical for *S. intercalatum* in one (3%) case; the species had not been specified/could not be identified in five cases (14%), which were identified by PCR only. Additionally, in one case (3%), the diagnosis of intestinal schistosomiasis was made from a biopsy of the patient’s appendix, but the species of *Schistosoma* could not be identified. The other 58 patients were diagnosed with urinary schistosomiasis (Supplementary Table S2) via identification of eggs with a shape typical of those of *S. haematobium* in filtered and/or sedimented urine in 57 cases (98%) and via detection of *Schistosoma* DNA by PCR (no species identification) in one (2%) case.

### Genetic profiling of *Schistosoma* infections

Mitochondrial (mt) *cox1*, nuclear ITS1 + 2 and/or 18S (mt/nuclear) genotypes were successfully obtained from 51 of the 94 (54%) samples analysed (Supplementary Table S2). For the remaining 43, only a partial mt genotype was obtained (Supplementary Table S2).

**Fig. 2:**
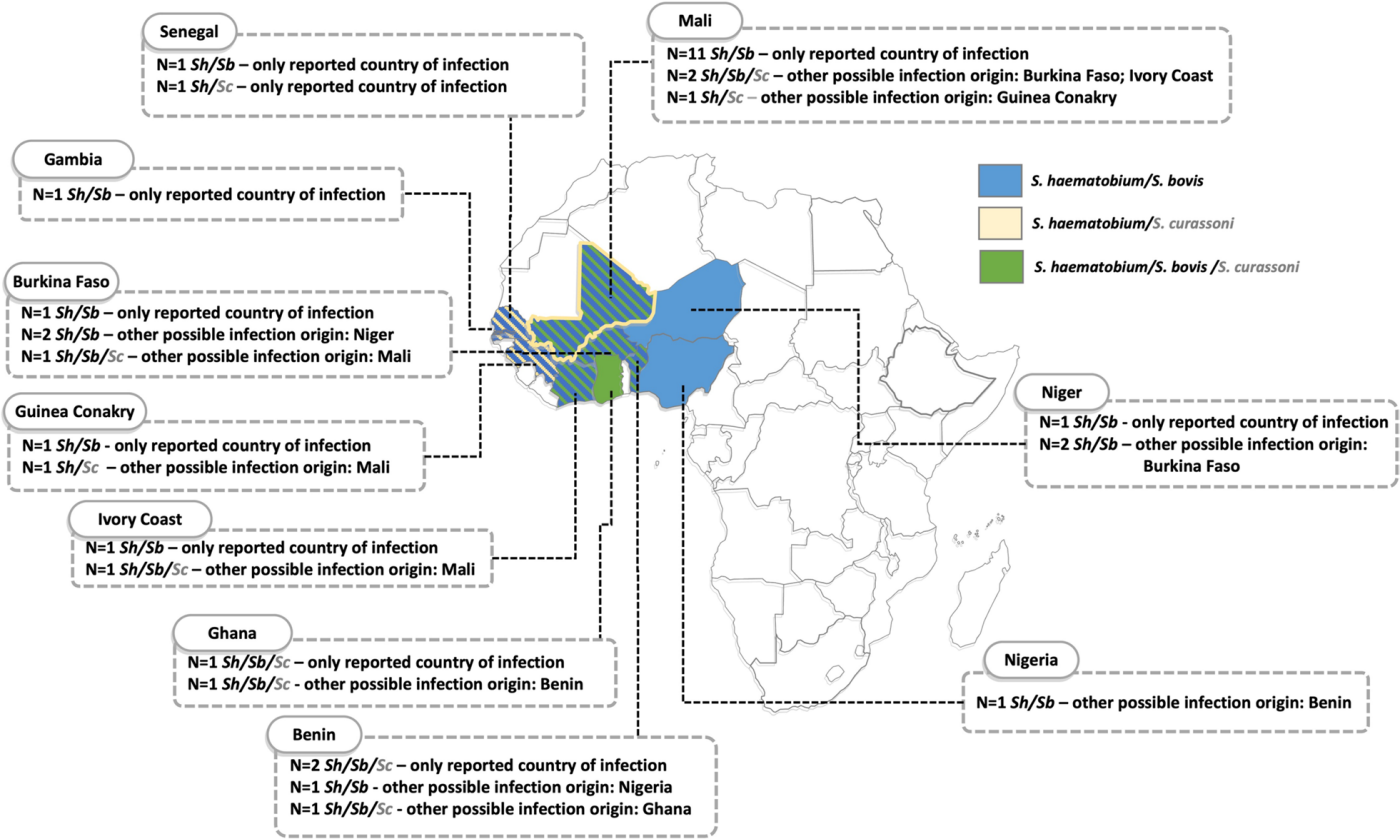
Country of origin and other reported possible origin(s) of infection in cases with mixed genetic profiles. Areas indicated in blue show the countries of possible infection for cases that presented *Schistosoma haematobium/Schistosoma bovis* mixed genetic profiles. Areas bordered in yellow show the countries of possible infection for cases that presented *Schistosoma haematobium/Schistosoma curassoni* mixed genetic profiles. Areas indicated in green show the countries of possible infection for cases that presented *S. haematobium/S. bovis/S. curassoni* mixed genetic profiles. A possible origin of infection with *S. curassoni* is indicated in grey due to the uncertainty of the identification (see “Discussion”). All infections were detected in urine with the exception of one case, which presented a mixed *S. haematobium/S. bovis* profile and was associated with a stool sample from a Senegalese patient who reported having travelled in Mali. The map was produced using www.yourfreetemplates.com. Individual patient’s data are available in Supplementary Table S2. *Sh* *Schistosoma haematobium*,* Sb* *S. bovis*, *Sc* *S. curassoni*

Cases with a diagnosis of intestinal or urinary schistosomiasis, but with identification of an atypical *Schistosoma* species (i.e. different from *S. mansoni* in intestinal infections and *S. haematobium* in urinary infections), are described in detail in Table 2, together with their genetic profile and recorded origin of infection (Fig. 2).

**Table 2:** Details of patients with mixed *Schistosoma* genetic profiles or infection with species not typical of human infection

Patient identifier (#ID)^a^	Original schistosomiasis diagnosis/material	Species identification by microscopy at diagnosis	*COX1*	ITS1 + 2	18S	Final identification	Country of birth	Other reported country/countries of possible infection
#2	Intestinal/stool	Sh	Sb	n.d.	Sh	Sh/Sb	Mali	–
#3	Urinary/urine	Sh	Sh/Sb	Sh	n.a.	Sh/Sb	Senegal	–
#6	Urinary/urine	Sh	Sh/Sb	Sh	n.a.	Sh/Sb	Mali	–
#7	Urinary/urine	Sh	Sh/Sb	Sh	n.a.	Sh/Sb	Mali	–
#15	Urinary/urine	Sh	Sh/Sb	Sh	n.a.	Sh/Sb	Mali	–
#23	Urinary/urine	Sh	Sb	Sh	n.a.	Sh/Sb	Mali	–
#34	Urinary/urine	Sh	Sh/Sb	Sh	n.a.	Sh/Sb	Niger	–
#41	Urinary/urine	Sh	Sh/Sb	Sh	n.a.	Sh/Sb	Mali	–
#42	Urinary/urine	Sh	Sh	Sh/Sc	Sh/Sc	Sh/Sc	Senegal	–
#52	Urinary/urine	Sh	Sb	Sh	n.a.	Sh/Sb	Mali	–
#53	Urinary/urine	Sh	Sb	Sh/Sc	n.d.	Sh/Sb/Sc	Ghana	–
#54	Urinary/urine	Sh	Sb	Sh/Sc	n.d.	Sh/Sb/Sc	Benin	–
#56	Urinary/urine	Sh	Sb	Sh	n.a.	Sh/Sb	Burkina Faso	Niger
#57	Urinary/urine	Sh	Sb	Sh	n.a.	Sh/Sb	Benin	Nigeria
#58	Urinary/urine	Sh	Sh/Sb	Sh	n.a.	Sh/Sb	Mali	–
#60	Urinary/urine	Sh	Sh	Sh/Sc	n.d.	Sh/Sc	Guinea Conakry	Mali
#61	Urinary/urine	Sh	Sb	Sh	n.a.	Sh/Sb	Guinea Conakry	–
#62	Urinary/urine	Sh	Sb	Sh	n.a.	Sh/Sb	Burkina Faso	Niger
#63	Intestinal/stool	*Si*	Sb	n.d.	n.d.	Sb	Senegal	Mali
#64	Urinary/urine	Sh	Sh/Sb	Sh/Sb	n.a.	Sh/Sb	Mali	–
#67	Urinary/urine	Sh	Sh/Sb	Sh/Sb	n.a.	Sh/Sb	Mali	–
#68	Urinary/urine	Sh	Sb	Sh	n.a.	Sh/Sb	Burkina Faso	–
#69	Urinary/urine	Sh	Sb	Sh/Sb/Sc	Sh/Sc	Sh/Sb/Sc	Burkina Faso	Mali
#71	Urinary/urine	Sh	Sh/Sb	Sh	n.a.	Sh/Sb	Mali	–
#72	Urinary/urine	Sh	Sh/Sb	Sh/Sb/Sc	Sh/Sc	Sh/Sb/Sc	Benin	Ghana
#73	Urinary/urine	Sh	Sh	Sh/Sb/Sc	Sh/Sc	Sh/Sb/Sc	Ivory Coast	Mali
#74	Urinary/urine	Sh	Sb	Sh	n.a.	Sh/Sb	Ivory Coast	–
#76	Urinary/urine	Sh	Sb	Sh/Sb/Sc	n.d.	Sh/Sb/Sc	Benin	–
#78	Urinary/urine	Sh	Sb	Sh	n.a.	Sh/Sb	Gambia	–

*Sh Schistosoma haematobium*, *Si Schistosoma intercalatum*, *Sb Schistosoma bovis*, *Sc Schistosoma curassoni*, *n.d.* not detected, *n.a.* not analysed,* COX1* cytochrome oxidase sub-unit 1,* ITS* internal transcribed spacer

^a^Patient #ID assigned in the raw data file (Supplementary Table S2)

Of the 36 cases clinically diagnosed as intestinal schistosomiasis, a mt/nuclear genotype was only obtained from two samples. For the remaining 34 samples, only the mt mitotype was obtained, so full identification was not possible. For the 32 (32/36; 89%) samples identified as containing eggs with the typical shape of those of *S. mansoni* at diagnosis, 31 (31/36; 86%) had a *S. mansoni cox1* mitotype and one had a *S. mansoni* mt/nuclear genotype (1/36; 3%). For the three samples clinically diagnosed as showing intestinal schistosomiasis but that contained eggs typical of *S. haematobium* or *S. intercalatum* (3/36; 8%), a mt/nuclear genotype was obtained from one sample and only the mt mitotype from the other two. The sample from which a full mt/nuclear genotype was obtained was from a patient from Mali; a mixed *S. haematobium/S. bovis* genotype was identified (*S. bovis cox1*, Sh 18S). For one case from Senegal, a *S. haematobium* mitotype was obtained. For the sample from a patient from Senegal, who had also travelled to Mali and was diagnosed with *S*. *intercalatum* infection based on egg morphology, a *S. bovis* mitotype was obtained. Finally, for the sample (1/36; 3%) from the Australian patient who had extensively travelled in Africa, and who had been diagnosed with schistosomiasis based on the presence of *Schistosoma* spp. eggs in an appendix biopsy, a *S. haematobium* mitotype was obtained.

Of the 58 patients clinically diagnosed with urinary schistosomiasis, full mt/nuclear genotypes were obtained from 49 samples, while only mt mitotypes were obtained from nine samples. Twenty-two samples (22/58; 38%) had *S. haematobium* mt/nuclear genotypes, while nine (9/58; 16%) had a *S. haematobium* mitotype. For 19 samples (19/58; 33%), a *S. haematobium/S. bovis* mt/nuclear genotype was obtained. For eight samples (8/58; 14%), the ITS1 + 2 analysis suggested the presence of S*. haematobium/S. curassoni* or *S. haematobium/S. bovis/S. curassoni*. However, the ITS1 + 2 rDNA marker contained only a single SNP in the ITS1 region that is thought to distinguish *S. bovis* and *S. curassoni*, which is not always sequenced to a very high standard due to its 5’ position. Therefore, to further clarify these nuclear genetic profiles, the partial 18S rDNA region was analysed; however, the 18S region could only be obtained from four of these samples. Two samples (2/58; 3%) were confirmed to have a *S. haematobium/S. curassoni* (one based on mt *cox1* and ITS1 + 2 + 18S and one based on mt *cox1* and ITS1 + 2) genotype, and six samples (6/58; 10%) were identified as having a *S. haematobium/S. bovis/S. curassoni* (three based on mt *cox1*, ITS1 + 2 + 18S and three based on mt *cox1* and ITS1 + 2) genotype (Table 2).

### Mitochondrial *cox1* phylogenetic analysis

**Fig. 3:**
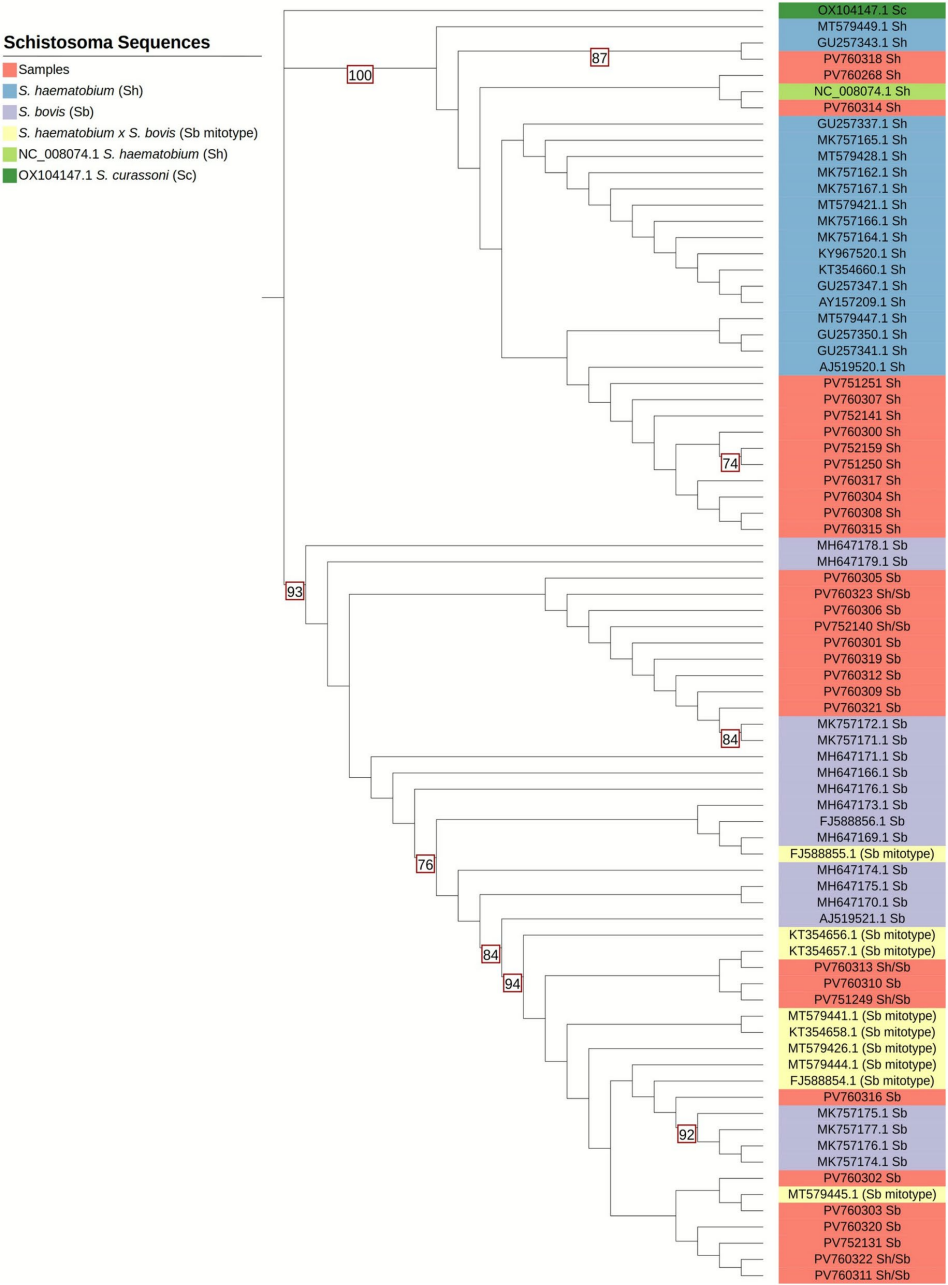
Phylogenetic tree based on partial cytochrome oxidase sub-unit 1 (*cox1*) sequences. Each sequence is colour-coded according to group [study samples with their GenBank accession numbers (red) and reference sequences (blue, yellow, purple, and light green) (*Schistosoma haematobium* NC_008074.1)]. To comprehensively visualize the phylogenetic placement of the *cox1* data from samples included in our study, and their relationships within the broader phylogenetic context, bootstrap support values ≥ 70% are displayed, as this threshold is used to indicate a minimum level of confidence and robustness in the phylogeny. Approximately 0.0034 substitutions per site across 155 tree branches showed high sequence similarity. The tree clearly shows two phylogenetic clusters associated with *Schistosoma haematobium* and *Schistosoma bovis* *cox1* data (both from hybrid and non-hybrid forms)*.* The *cox1* data obtained from this study fall into these two clusters, based on the *cox1* mitotype obtained from each sample; *S. haematobium/S. bovis* hybrid forms, which present a *S. bovis* mitotype cluster with the *S. bovis cox1* cluster, and all samples that presented a *S. haematobium* mitotype cluster with the *S. haematobium cox1* cluster. The *cox1* analysis alone cannot distinguish hybrid and non-hybrid forms or infer the origin of the infection. *Schistosoma curassoni* OX104147.1 *cox1* data (dark green) were used as the outgroup

The phylogenetic analysis was performed on the available (38/94, 41%) *cox1* (~ 956 bp) sequences from the study samples; however, the sequences of six samples (#24, #41, #64, #68, #70, #81) were eventually excluded due to low quality bases, resulting in the final inclusion of 32 *cox1* sequences (Fig. 3). Selected *S. haematobium, S. bovis* and *S. bovis* mitotype (mtDNA haplotype) from *S. haematobium* × *S. bovis* hybrids were also included, and *S. curassoni* OX104147.1 was used as the outgroup.

The phylogenetic tree (Fig. 3) had a mean length of approximately 0.0034 substitutions per site across 155 branches, indicating high sequence similarity. Samples identified as *S. haematobium* (having both a *S. haematobium* ITS1 + 2 and *cox1* genotype) clustered in well-supported branches, having generally a bootstrap > 70% with respect to other published *S. haematobium* representative sequences. Samples identified as having mixed species genetic profiles (*S. bovis cox1* and *S. haematobium* ITS1 + 2 genotype) (Table 2) clustered with *S. bovis* or *S. haematobium* × *S. bovis* (*S. bovis* mitotypes) representative *cox1* sequences, supporting their identification.

## Discussion

A high prevalence of *Schistosoma* infection in migrants entering Europe has been reported in a number of studies [6], and recent events of autochthonous transmission of urinary schistosomiasis in Spain and Corsica [11, 23–28] have shown the potential for *Schistosoma* species to colonize new environments. These findings highlight the need for coordinated efforts and vigilance with respect to the possible introduction and establishment of schistosomes and their autochthonous transmission, and prompt diagnosis and effective treatment of infected patients. The present study explored the geographical origin and genetic profiles of *Schistosoma* infections of migrants and travellers travelling to Europe from Africa and diagnosed within a network of 11 European centres specializing in traveller and migrant health.

Our results, obtained from a large cohort of patients, show that while infection with *S. haematobium* and *S. mansoni* were identified in the majority of cases (66/94; 70%), infections with mixed *Schistosoma* spp. genetic profiles represented a large proportion of cases being diagnosed in Europe. Mixed genetic profiles were identified in at least 30% (28/94) of the samples, and were almost exclusively (27/28; 96%) associated with cases of urinary schistosomiasis. Only one mixed genetic profile (*S. haematobium/S. bovis*) was diagnosed among the cases of intestinal schistosomiasis, while among the urinary infections, almost half (27/58; 47%) could be identified as having a mixed genetic profile. The majority of samples with mixed genetic profiles (26/28; 93%) included genetic traits of *S. haematobium* and *S. bovis*. Furthermore, all samples with mixed genetic profiles were from patients who reported probable infection in a West African country. These results are overall in line with those reported by Salas-Coronas et al. [15], who found a prevalence of 39% for infections that presented mixed *Schistosoma* genetic profiles, most commonly *S. haematobium/S. bovis*, from a total of 31 migrants from sub-Saharan Africa diagnosed with urogenital schistosomiasis at a single centre in Almería, Spain. We did not find a single *S. haematobium/S. mansoni* profile, contrary to Salas-Coronas et al. [15] and De Elias-Escribano et al. [33], who identified this hybrid type in a high proportion of their samples based on the analysis of a single egg/miracidia. These differences could be due to the fact that ITS amplification could not be achieved for many of the samples in our study, especially for stool samples, which are associated with *S. mansoni*. This could be related to the stool being a complex matrix containing PCR inhibitors, which are not always removed completely during DNA extraction procedures. This, together with the long length of the ITS1 + 2 fragment (~ 981 bp) can result in difficulties in amplification and in achieving high-quality Sanger sequence data. As a result, epidemiological studies such as this one could be biased towards the identification of genetic heterogeneity in urogenital compared to intestinal schistosomiasis, which may lead to unbalanced identification and characterization of human infections.

Additionally, in the present study, we identified dual *S. haematobium/S. curassoni* profiles in two samples and triple *S. haematobium/S. bovis/S. curassoni* profiles in six samples. However, it must be noted that the ITS1 + 2 rDNA marker cannot be easily used to distinguish between *S. bovis* and *S. curassoni*, and that four out of these eight samples were identified based only on *cox1* and ITS1 + 2 rather than on the combination of *cox1*, ITS and 18S, which provides more robust nuclear profiling of samples. The mixed profiles that include *S. curassoni* originated from patients who were infected within a possibly wider area (Benin, Burkina Faso, Ghana, Guinea, Ivory Coast, Mali, Senegal), compared to that reported so far (Ivory Coast, Mali, Mauritania, Niger, Nigeria, Senegal) [13, 15, 38, 39]. However, one drawback of evaluating imported infections is that the geographical origin of infection might not be completely certain due to recall bias and because many migrants endure a long journey through multiple countries endemic for *Schistosoma* before arriving in Europe. This adds further to inaccuracies deriving from the fact that countries of potential infection, in this study, were identified retrospectively based on the travel history collected during the routine clinical visit, which may have been incomplete or inaccurate.

Migrants infected with *Schistosoma* spp. are often diagnosed and treated long after they have arrived in Europe, potentially with severe consequences for their health [29, 30], which again stresses the need for prompt diagnosis and treatment. From a clinical perspective, the impact of *Schistosoma* co-infections and/or hybrids/introgressed forms on clinical manifestation is the subject of much speculation regarding possible changes in the observed clinical picture due to an increased occurrence of these forms. However, the studies that have been carried out so far present inconclusive results [15, 16]. Furthermore, the overall results in the literature seem to point towards the hybridization that is detected today as being mostly the result of “ancient” episodes with de novo spillover from animals being a rare phenomenon [10, 12, 40, 41]. This allows us to speculate that the spectrum of pathological presentations that we will face in the clinical setting in the near future, and thus the approach to treatment, will probably tend to remain stable. However, a possible future increase in co-infections due to environmental changes warrants vigilance. Unfortunately, due to the study design, which relied on samples and data obtained from routine clinical records, we could not undertake a correlation analysis of the genetic profiles and clinical manifestations.

This study has several limitations, in addition to the above-mentioned uncertainties regarding the origins of the infections and the definitive identification of the genetic profiles of some of the samples. First, the study was conducted on stored samples made available through routine practice, thus the results might not accurately reflect the true relative prevalence of each *Schistosoma* genetic profile in infections in migrants and travellers. Second, as mentioned above, we carried out molecular analyses of, essentially, a pool of eggs from each sample, allowing the identification of DNA from each species in the different samples but not the genetic profiles of individual schistosomes. Therefore, we cannot reach a definitive conclusion as to whether the mixed genetic profiles found were derived from co-infections or hybrid forms, or the coexistence of both. Furthermore, the identification of almost all “pure” *S. mansoni* infection and about one-fourth of the “pure” * S. haematobium* infections were based only on mt *cox1* data, reducing our ability to infer the true level of mixed genetic profiles in the majority of the samples. Third, although the samples were from patients who could have been infected in a wide range of countries, these were all sub-Saharan countries, and mainly in West Africa. Thus, samples from East Africa were underrepresented and samples from South America were not investigated. Fourth, due to the nature of this study, which relied on samples and data obtained from routine clinical records, we could not undertake a correlation of the genetic profiles and clinical pathology. Also, we could not be sure if the presence of *S. haematobium* (± *S. bovis*) in some stool samples was due to urine contamination rather than the result of intestinal (mesenteric vein) infection. In any case, the retrieval of *S. haematobium* eggs in an appendix biopsy shows that infection by this species in the mesenteric veins is possible. Additionally, detailed information on the morphology of the eggs was not available from the clinical diagnoses, therefore we could not correlate any of our data with  microscopical observations. However, our data confirm the largely acknowledged finding that the morphology of eggs alone is inadequate for the definitive identification of *Schistosoma* species [18].

## Conclusions

Our results, which were obtained from a large cohort of imported *Schistosoma* cases from sub-Saharan Africa, showed that infections with *S. haematobium* and *S. mansoni* represent the majority of cases being diagnosed in Europe; however, mixed *Schistosoma* genetic profiles (mostly *S. haematobium/S. bovis*) were identified in at least 30% of samples. Our results call for a coordinated effort for the prompt diagnosis of *Schistosoma* infections in migrants and travellers , so that appropriate treatment and case management can be promptly implemented, together with monitoring of the possible introduction of *Schistosoma* species and the establishment of their autochthonous transmission where compatible snail intermediate hosts exist and human contact with waterbodies is common. In high-income countries, such as those in Europe where sanitation is generally of a high standard, the introduction of *Schistosoma* parasites causing urinary infection (*S. haematobium*) might be more relevant than the introduction of *S. mansoni*, since the intermediate hosts of the former, snails of the genus *Bulinus*, are present in Europe [18, 32] and contamination of freshwater is more likely to occur via open urination than via open defecation. However, vigilance with respect to all *Schistosoma* spp. introduced into Europe is warranted.

## Supplementary Information

Additional file 1: Figure S1. Molecular analyses flowchart.

Additional file 2: Table S1. Primers for *Schistosoma* detection.

Additional file 3: Table S2. Raw data file.

Additional file 4: Text S1. Details of ethics approval of the study and use of samples from the participating centres.

## Data Availability

All of the data used in this study and the GenBank accession numbers have been made available in Supplementary Table S2.

## Abbreviations

*cox1*: Cytochrome oxidase sub-unit 1
ITS: Internal transcribed spacer
