# Supplementary figures and images for: Investigating genetic profiles of cases of Schistosoma spp. imported into Europe: a cohort from the European Society of Clinical Microbiology and Infectious Diseases Study Group for Clinical Parasitology
Source: Parasit Vectors. 2025 Dec 15;19:37. doi: 10.1186/s13071-025-07164-5 (PMC12822218; doi:10.1186/s13071-025-07164-5)

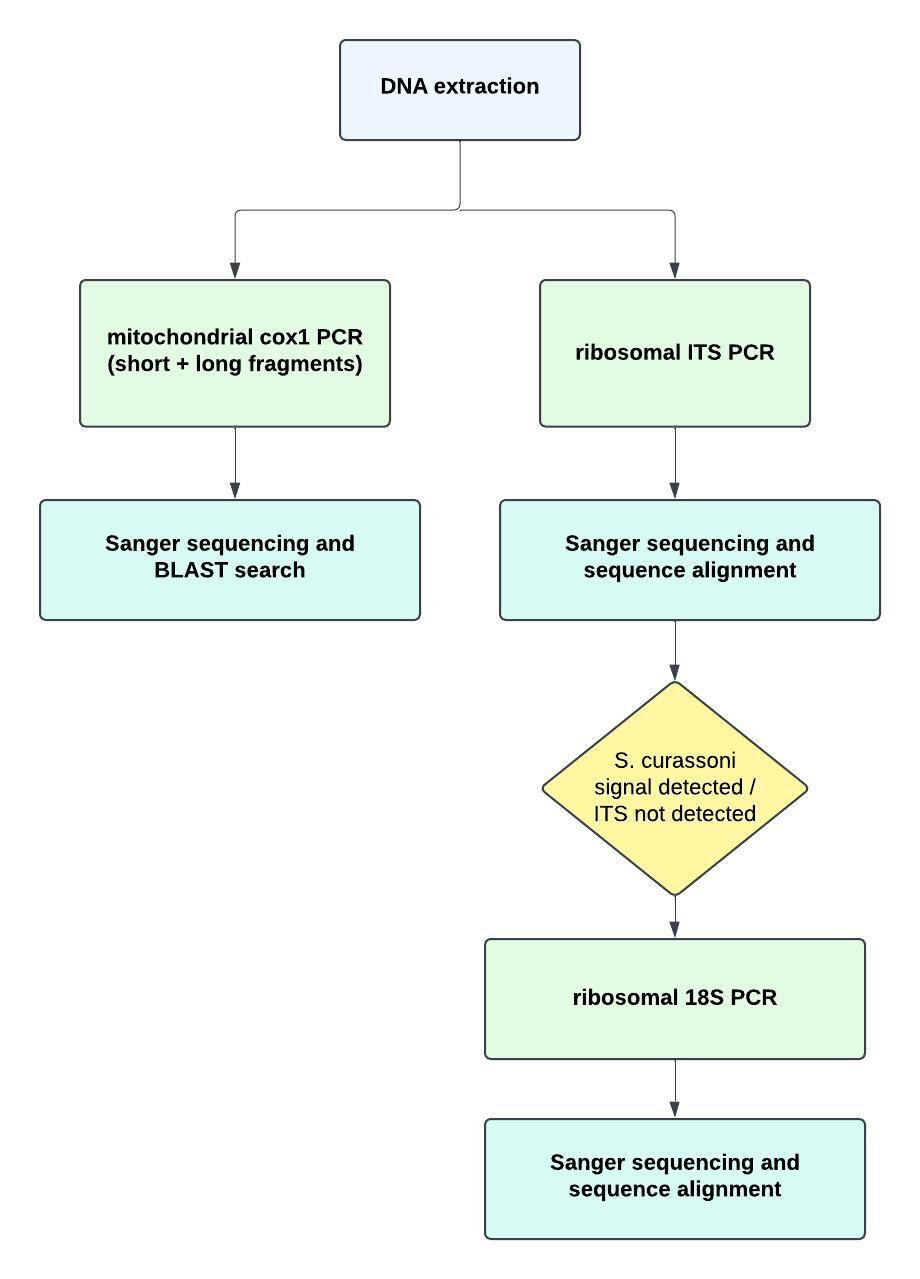

Supplement: Supplementary file 1 — Additional file 1: Figure S1. Molecular analyses flowchart. [file 13071_2025_7164_MOESM1_ESM.jpeg]
