# Supplementary material for: Investigating genetic profiles of cases of Schistosoma spp. imported into Europe: a cohort from the European Society of Clinical Microbiology and Infectious Diseases Study Group for Clinical Parasitology
Source: Parasit Vectors. 2025 Dec 15;19:37. doi: 10.1186/s13071-025-07164-5 (PMC12822218; doi:10.1186/s13071-025-07164-5)
Supplement: Supplementary file 2 — Additional file 2: Table S1. Primers for Schistosoma detection. [file 13071_2025_7164_MOESM2_ESM.docx]

**Table S1.** Primers for *Schistosoma* detection.

| **Target** | **Oligonucleotide** | **Sequence (5’ → 3’)** | **Amplicon length (bp)** | **Reference** |
| --- | --- | --- | --- | --- |
| *cox1*  (short fragment) | Asmit1_F  Sh_R  Sb_R  Sman_R | TTTTTTGGTCATCCTGAGGTGTAT  TGATAATCAATGACCCTGCAATAA  CACAGGATCAGACAAACGAGTACC  TGCAGATAAAGCCACCCCTGTG |  | [1,2] |
|  |  |  | ~543 |  |
|  |  |  | ~306 |  |
|  |  |  | ~375 |  |
| *cox1*  (long fragment) | COX1Schisto5_F  COX1Schisto3_R | TCTTTRGATCATAAGCG  TAATGCATMGGAAAAAAACA | ~956 | [3] |
| ITS1+2 | ITTS2_F  ITTS1_R | AACAAGGTTTCCGTAGGTGAA  TGCTTAAGTTCAGCGGGT | ~981 | [1] |
| 18S | 18S_WA_F  18S_300_R | GCGAATGGCTCATTAAATCAG  TCAGGCTCCCTCTCCGGA | ~289 | [1] |

[1] Pennance T, Allan F, Emery A, Rabone M, Cable J, Garba AD, Hamidou AA, Webster JP, Rollinson D, Webster BL. Interactions between Schistosoma haematobium group species and their Bulinus spp. intermediate hosts along the Niger River Valley. Parasit Vectors. 2020 May 24;13(1):268. doi: 10.1186/s13071-020-04136-9

[2] Schols R, Carolus H, Hammoud C, Mulero S, Mudavanhu A, Huyse T. A rapid diagnostic multiplex PCR approach for xenomonitoring of human and animal schistosomiasis in a 'One Health' context. Trans R Soc Trop Med Hyg. 2019 Nov 1;113(11):722-729. doi: 10.1093/trstmh/trz067.

[3] Webster BL, Emery AM, Webster JP, Gouvras A, Garba A, Diaw O, Seye MM, Tchuente LA, Simoonga C, Mwanga J, Lange C, Kariuki C, Mohammed KA, Stothard JR, Rollinson D. Genetic diversity within Schistosoma haematobium: DNA barcoding reveals two distinct groups. PLoS Negl Trop Dis. 2012;6(10):e1882. doi: 10.1371/journal.pntd.0001882
