# Supplementary material for: Investigating genetic profiles of cases of Schistosoma spp. imported into Europe: a cohort from the European Society of Clinical Microbiology and Infectious Diseases Study Group for Clinical Parasitology
Source: Parasit Vectors. 2025 Dec 15;19:37. doi: 10.1186/s13071-025-07164-5 (PMC12822218; doi:10.1186/s13071-025-07164-5)
Supplement: Supplementary file 4 — Additional file 4: Text S1. Details of ethics approval of the study and use of samples from the participating centres. [file 13071_2025_7164_MOESM4_ESM.docx]

**Additional File S1. Details of ethics approval of the study and use of samples from the participating centers.**

Erasmus MC University Medical Centre Rotterdam (Rotterdam, The Netherlands; Erasmus MC MEC 2012-047); Hospital Universitari Vall d’Hebron (Barcelona, Spain; PR(AG)344/2021, 06-08-2021); University Hospital, LMU Munich (Munich, Germany; 21-0554); Azienda Ospedaliera Universitaria Careggi (Firenze, Italy; BIO_25910, 27-02-2024); Swiss Tropical and Public Health Institute (Basel, Switzerland; EKNZ UBE-15/22, EKNZ Req-2016-00050); ISGlobal Hospital Clinic Barcelona (Barcelona, Spain; HCB/2025/002); Université Sorbonne Paris Nord, Hôpital Avicenne (Comité Local d' Ethique pour la recherche clinique des HUPSSD Avicenne, France; Protocole Numero: CLEA-2025-478, 30/06/2025); Leiden University Medical Center (Leiden, The Netherlands; LUMC Review Committee Biobank & biomaterials [TCBio] February 29, 2024); Institute of Tropical Medicine Antwerp (Antwerp, Belgium; IRB n. 1509/21, 18/06/2021); Hospital Universitario La Paz Carlos III (Madrid, Spain; PI-4934, 23-09-2021).
